# Identification of Immunodominant Antigens From a First-Generation Vaccine Against Cutaneous Leishmaniasis

**DOI:** 10.3389/fimmu.2022.825007

**Published:** 2022-05-12

**Authors:** María José Germanó, Juan Pablo Mackern-Oberti, Jessica Gardone Vitório, Mariana Costa Duarte, Daniel Carvalho Pimenta, Maria Victoria Sanchez, Flavia Alejandra Bruna, Esteban Sebastián Lozano, Ana Paula Fernandes, Diego Esteban Cargnelutti

**Affiliations:** ^1^ Instituto de Medicina y Biología Experimental de Cuyo (IMBECU), Consejo Nacional de Investigaciones Científicas y Técnicas (CONICET), Universidad Nacional de Cuyo (UNCuyo), Mendoza, Argentina; ^2^ Facultad de Ciencias Médicas (FCM), Universidad Nacional de Cuyo (UNCuyo), Mendoza, Argentina; ^3^ Departamento de Análises Clínicas e Toxicológicas, Faculdade de Farmácia, Universidade Federal de Minas Gerais, Belo Horizonte, Brazil; ^4^ Laboratório de Bioquímica e Biofísica, Instituto Butantan, São Paulo, Brazil

**Keywords:** *L. amazonensis*, immunoproteomic analysis, vaccines, American tegumentary leishmaniasis, neglected tropical disease (NTD)

## Abstract

Leishmaniasis is a neglected tropical disease (NTD) caused by parasites belonging to the *Leishmania* genus for which there is no vaccine available for human use. Thus, the aims of this study are to evaluate the immunoprotective effect of a first-generation vaccine against *L. amazonensis* and to identify its immunodominant antigens. BALB/c mice were inoculated with phosphate buffer sodium (PBS), total *L. amazonensis* antigens (TLAs), or TLA with Poly (I:C) and Montanide ISA 763. The humoral and cellular immune response was evaluated before infection. IgG, IgG1, and IgG2a were measured on serum, and IFN-γ, IL-4, and IL-10 cytokines as well as cell proliferation were measured on a splenocyte culture from vaccinated mice. Immunized mice were challenged with 10^4^ infective parasites of *L. amazonensis* on the footpad. After infection, the protection provided by the vaccine was analyzed by measuring lesion size, splenic index, and parasite load on the footpad and spleen. To identify immunodominant antigens, total proteins of *L. amazonensis* were separated on 2D electrophoresis gel and transferred to a membrane that was incubated with serum from immunoprotected mice. The antigens recognized by the serum were analyzed through a mass spectrometric assay (LC-MS/MS-IT-TOF) to identify their protein sequence, which was subjected to bioinformatic analysis. The first-generation vaccine induced higher levels of antibodies, cytokines, and cell proliferation than the controls after the second dose. Mice vaccinated with TLA + Poly (I:C) + Montanide ISA 763 showed less footpad swelling, a lower splenic index, and a lower parasite load than the control groups (PBS and TLA). Four immunodominant proteins were identified by mass spectrometry: cytosolic tryparedoxin peroxidase, an uncharacterized protein, a kinetoplast-associated protein-like protein, and a putative heat-shock protein DNAJ. The identified proteins showed high levels of conserved sequence among species belonging to the *Leishmania* genus and the Trypanosomatidae family. These proteins also proved to be phylogenetically divergent to human and canine proteins. TLA + Poly (I:C) + Montanide ISA 763 could be used as a first-generation vaccine against leishmaniasis. The four proteins identified from the whole-protein vaccine could be good antigen candidates to develop a new-generation vaccine against leishmaniasis.

## 1 Introduction

Leishmaniasis is a parasitic disease caused by protozoa belonging to the *Leishmania* genus, which is considered to be a neglected tropical disease (NTD) by the World Health Organization (WHO) (1). This disease is distributed throughout 98 countries, including 20 countries of the American continent, such as Brazil and Argentina (1). There are different clinical manifestations of leishmaniasis, depending on the *Leishmani*a species, parasite load, and the immunological, nutritional, and genetic background of the host (2, 3). The clinical forms of the disease include cutaneous, mucocutaneous, and visceral leishmaniasis. There are also several other forms that can be considered collectively as tegumentary leishmaniasis (4). In Argentina, there are four species responsible for the clinical manifestations: *L. amazonensis*, *L. braziliensis*, *L. guyanensis*, and *L. infantum* (5). *L. amazonensis* is a relevant species in the American continent, which is mainly responsible for the more severe forms of cutaneous clinical manifestation, such as diffuse cutaneous leishmaniasis (6).

Currently, there are no licensed vaccines available for human use that prevent leishmaniasis (7). Even though there are three commercial vaccines available against canine leishmaniasis (8), these would not necessarily have the same effectiveness against leishmaniasis in all countries of the world. It could be due to the complex immune response elicited by different *Leishmania* species (9, 10), which are not distributed equally in geographical terms (11). In addition, they would also require adequation for human use, including changes to the adjuvants (12).

The use of adjuvants in vaccines is very important to increase and modulate the immune response towards an appropriate profile. Polyinosinic–polycytidilic acid [Poly (I:C)] is a Toll-like receptor 3 (TLR-3) agonist that elicits both Th1 innate and adaptive immunity (13). Hence, this molecule induces IFN production and upregulates the co-stimulatory and activation markers such as CD86, CD40, and MHC class II in an IFN-dependent manner (14). Furthermore, it has been demonstrated that Poly (I:C) can induce the cross-presentation of antigens by DC and, consequently, enhance the CD8+ T-cell response, which is important to control intracellular parasitic infection (15). Our research group has previously demonstrated that total *L. amazonensis* antigens (TLAs), obtained by cycles of freezing and thawing or sonication, combined with Poly (I:C) as adjuvant, were able to partially protect BALB/c mice against the *L. amazonensis* infection (16, 17).

However, nucleotide acid-based adjuvants, such as Poly (I:C), are unstable due to their degradation by nucleases (14). As a result, the use of particulate formulations, such as emulsions, could be a good strategy to solve this problem (18). Montanide ISA 763 is a water-in-oil emulsion that generates a depot in the site of inoculation, allowing a slow release of the antigen. Also, this emulsion protects antigens from enzymatic degradation; modifies its electric charge, becoming more immunogenic; and improves the antigen uptake by antigen-presenting cells (APCs) (19). Our group has demonstrated that this oleaose adjuvant was effective against *L. amazonensis* infection when it was combined with TLA obtained by cycles of freezing and thawing (20). In addition, the efficacy of the use of Montanide as an adjuvant in vaccines against cutaneous leishmaniasis has been demonstrated by other authors, with more conclusive reults than the BCG adjuvant (21, 22). Consequently, the combination of Poly (I:C), as a TLR-3 ligand, with Montanide ISA 763, as water-in-oil emulsion, may prevent both the antigen and immunostimulant adjuvant from degradation and enhance the efficacy of the vaccine against leishmaniasis.

Although the development of first-generation vaccines is an economic strategy in order to enable production in low-income countries, they are unstable, they present problems associated with standardization, and there is also a lack of knowledge about their composition (23). One-protein-based vaccines could solve this problem, but the antigen selection is the main point to be considered. A successful approach used to identify the best antigen candidates for vaccines against pathogens is the use of immunoproteomics, which is based on a two-dimensional electrophoresis followed by Western blotting (24–26).

In this study, a reverse vaccinology approach has been applied to identify *L. amazonensis* protective antigens. First, TLA formulated with Poly (I:C) has been emulsified in Montanide ISA 763 to evaluate their immune response and protective effect against the experimental infection by *L. amazonensis* in a mouse model. After demonstration of efficacy as indicated by low parasite loads and decreased lesion size in vaccinated animals, serum from immunoprotected mice was used to identify protective immunodominant antigens by immunoproteomics.

## 2 Materials and Methods

### 2.1 Animals

Six- to 8-week-old female BALB/c mice were used to perform two independent experiments. All animals were kept under standard conditions (barriers, 12-h light cycle, controlled room temperature, and water and food provided *ad libitum*). All animals were cared for in accordance with the Core Principles for the Care and Use of animals in research by the NIH. All procedures performed in studies involving animals were approved by the Institutional Animal Care and Use Committee of the School of Medical Science, Universidad Nacional de Cuyo (protocol approval no. 80/2016).

### 2.2 Vaccine Formulations and Immunization

TLAs were obtained as previously reported (17). *L. amazonensis* (MHOM/VE/84/MEL) promastigotes were cultured until their late logarithmic phase in RPMI-1640 supplemented with 20% fetal bovine serum (FBS) and antibiotics (100 μg/ml streptomycin and 100 IU/ml ampicillin). Promastigotes were washed three times with phosphate buffer sodium (PBS) and disrupted by 5 cycles of freezing (-80°C) and thawing (56°C). The concentration of proteins was measured by bicinchoninic acid assay according to the manufacturer’s instructions (Thermo Scientific).

One hundred micrograms of TLA was formulated with 50 μg of Poly (I:C) and Montanide ISA 763 in a 70:30 (v/v) relation up to a final volume of 200 μl.

In order to analyze the immune response conferred by the first-generation vaccine, 5 animals per group were inoculated in a homologous prime/boost scheme with 2 doses every 21 days between them. The animal groups were as follows: PBS, TLA, and TLA + Poly (I:C) + Montanide ISA 763.

### 2.3 Humoral and Cellular Immune Response

Twenty-one days after each immunization, blood samples were obtained from twice-immunized mice. Anti-TLA IgG antibodies were determined 21 days after prime and boost, while anti-TLA IgG1 and IgG2a isotypes were determined 21 days after boost by enzyme-linked immunosorbent assay (16). Thus, 96-well plates were incubated with 3 μg/well of TLA overnight at 4°C. Unspecific sites were blocked with blocking buffer (PBS with 5% semi-skimmed milk) for 30 min at 37°C. Plates were incubated with serum samples (1:500 or 1:1,000 dilution to IgG or IgG isotype determination) for 1 h at 37°C. Secondary horseradish peroxidase-conjugated IgG antibody at 1:10,000, biotin-conjugated IgG1 at 1:2,000, or IgG2a (BD Pharmingen) at 1:1,000 in blocking buffer was added and the plates were incubated for 1 h at 37°C. For biotin-conjugated antibodies, plates were incubated with HRP-conjugated streptavidin at 1:2,000 in blocking buffer for 1 h at 37°C. Plates were incubated with 50 μl/well of tetramethylbenzidine (TMB, BD Bioscience) for 30 min, and the reaction was stopped with 2 N sulfuric acid. Absorbances were measured at 450 nm in an ELISA-plate reader (Thermo Scientific). Results were presented as the mean of optical density (O.D.) value + the standard error of the mean (SEM).

Twenty-one days after being boosted, the mice were euthanized, their spleens were removed and homogenized, and their red blood cells were lysed with ammonium chloride-potassium buffer (ACK, pH 7) and resuspended in RPMI-1640 media supplemented with 10% BSF, antibiotics, and β-mercaptoethanol (1:280,000). Splenocytes were plated in 96-well plates at 5 × 10^5^ cells/well in triplicate. Splenocytes were incubated with supplemented media (unstimulated control) or 1 μg/well TLA. Plates were incubated for 72 h at 37°C, 5% CO_2_ in a humid atmosphere.

Supernatants were recovered to determine IFN-γ, IL-4, and IL-10 cytokines by sandwich ELISA, according to the manufacturer’s instructions (BD Pharmingen).

After 72 h of incubation, cells were used to determine cell proliferation by Thiazolyl Blue Tetrazolium Bromide (MTT) assay (27). With that purpose, cells were incubated with 0.5 mg/ml of MTT (Sigma) for 4 h. Supernatant was removed, and formazan crystals were dissolved with 50 μl/well of dimethyl sulfoxide (DMSO). Absorbances were measured at 570 nm in an ELISA-plate reader (Thermo Scientific). Cell proliferation index was calculated according to the following formula:

PI = (OD value from stimulated cell)/(OD value from unstimulated cell).

### 2.4 Challenge and Infection Outcome

To analyze the protection outcome given by the first-generation vaccine, 5 animals per group were inoculated three times every 14 days with the same vaccine formulation. Fourteen days after the last immunization, animals were infected with 10^4^ late-stationary-phase *L. amazonensis* promastigotes on the right footpad. The protection outcome given by the vaccine was analyzed by different parameters: footpad swelling, splenic index, and parasite load on the footpad and spleen.

Footpad lesion was measured weekly over an 11-week period using a digital caliper (SCHWYZ, ED-10P). The value of each uninfected footpad was subtracted from each infected footpad.

Eleven weeks after infection, the mice were weighed and then euthanized. Their right foot and their spleen were removed and weighed. Splenic index was calculated according to the following formula:

Splenic index = (body weight/spleen weight) × 100

Parasite load was evaluated in the infected footpad and spleen. Therefore, the spleen and the infected footpad were homogenized in RPMI-1640 media supplemented with 20% FBS and antibiotics. Obtained suspensions were incubated in 96-well plates, and a 10-fold dilution was performed. After 14 days of incubation, wells were observed under an inverted microscope and the number of parasites was determined by the highest dilution where viable parasites were observed (28).

### 2.5 Identification of Immunodominant Antigens

To identify immunodominant antigens, total proteins of *L. amazonensis* were separated on 2D electrophoresis gel and transferred to a membrane that was incubated with serum from mice inoculated with PBS (negative control) and first-generation vaccine [TLA + Poly (I:C) + Montanide ISA 763]. Some recognized proteins by serum were analyzed by liquid chromatography–ion-trap time-of-flight tandem mass spectrometric assay (LC-MS/MS-IT-TOF) to identify their protein sequence.

#### 2.5.1 Total Protein Extraction

*L. amazonensis* promastigotes were cultured in RPMI-1640 supplemented with 20% FBS and antibiotics until their early-stationary growth phase. Dead parasites were removed by centrifugation at 50 × *g* for 10 min at 4°C, and 10^9^ live promastigotes were washed three times with PBS pH 7 (1,500 × *g* for 10 min at 4°C). The obtained pellet was resuspended in 1 ml of TNE buffer (50 mM Tris-HCl, pH 7.4, 150 mM NaCl, and 5 mM EDTA) with protease inhibitors (Sigma P2714). Samples were frozen 5 times with N liquid and thawed at 56°C, and proteins were precipitated with trichloroacetic acid (TCA, 10%). The obtained pellet was washed with acetone and resuspended in a 2D gel rehydration buffer [7 M urea, 2 M thiourea, and 4% CHAPS (Ludwig Biotec)]. Proteins were quantified with a 2D-Quanti kit (GE Healthcare), and their integrity was corroborated by sodium dodecyl sulfate–polyacrylamide gel electrophoresis (SDS-PAGE).

#### 2.5.2 Isoelectric Focusing

For every 250 µg of protein, 1.5% of immobilized pH gradient buffer (IPG), 20 mM dithiothreitol (DTT), and rehydration buffer up to a final volume of 125 µl were added.

Isoelectric focusing strips (4 cm, pH 4–7, Ready IPG Strip, Bio-Rad) were rehydrated with samples for 24 h at 20°C. They were isoelectronic focused using an Ettan Multiphor 3 system covered with PlusOne dry strip cover fluid (GE Healthcare), under the following conditions: 200 Vh 300 V step (STP), 300 Vh 1,000 V gradient (GRD), 4,800 Vh 5000 V GRD, 3,000 Vh 5,000 V STP, 18 h 300 V STP, and 1 h 5,000 V STP.

#### 2.5.3 Electrophoresis (Second Dimension) and Western Blot

After IEF, each strip was incubated for 15 min with 50 mM Tris-HCl buffer (pH 8.8), 6 M urea, 30% (v/v) glycerol, 2% (p/v) SDS, 0.002% bromophenol blue (p/v), and 1% (p/v) of dithiothreitol (DTT) in order to disrupt disulfide bridges. Then, a second incubation of 15 min was carried out with the same buffer, but replacing DTT with 125 mM iodoacetamide, in order to acetylate the thiol groups. Strips were placed in 12% polyacrylamide gel with SDS and sealed with agarose, bromophenol blue, and tris-glycine. A molecular weight marker (Bio-Rad Precision Plus Protein Standards Dual Color) was used, and the electrophoresis was run at 30 mA/gel in a Mini-PROTEAN ^®^ II system (Bio-Rad) until the running front reached the bottom.

Three gels were fixed for 2 h with a solution containing 10% acetic acid and 40% ethanol, and then incubated for 30 h with a solution containing colloidal Coomassie Brilliant Blue G-250 and 20% methanol. Later, the gels were distained with 20% methanol for 1.5 h and stored at 4°C in 10% ammonium bicarbonate. Images were obtained using the Chemi-Doc Imaging System (Bio-Rad).

Proteins from other gels were transferred to a nitrocellulose membrane at 250 mA for 2 h using Mini Protean II equipment. Unspecific sites were blocked with 5% skimmed milk in PBS for 2 h. Then, membranes were incubated for 2 h with a pool of serum (dilution 1:400 in 5% BSA, PBS, and 0.1% Tween 20) from mice immunized twice with PBS or TLA + Poly (I:C) + Montanide ISA 763. After that, membranes were incubated for 2 h at room temperature with anti-mouse IgG (dilution 1:5,000 in 5% BSA, PBS, and 0.1% Tween 20, Sigma). Finally, the membranes were revealed with a chemiluminescent reaction (Bio Rad Claruty Wester ECL), and the images were acquired using the Chemi-Doc Imaging System (Bio-Rad).

#### 2.5.4 Protein Digestion

Images obtained from membranes and gels were merged using Image Master Platinum software (GE HealthCare). Thirteen spots were identified, excised, destained with 50% acetonitrile and 25 mM ammonium bicarbonate pH 8, and dehydrated with 50% acetonitrile. Additionally, samples from a molecular weight marker and gel without protein were used as positive and negative controls.

After evaporating the remaining liquid with SpeedVac (AQ-Vac Eppendorf concentrator plus), proteins were digested by incubation for 16 h at 37°C with 10 µl of 20 µg/ml trypsin from porcine pancreas (Sigma) and 50 µl of 25 mM ammonium bicarbonate, pH 8. Peptides were obtained by incubation for 15 min with 30 µl of a solution containing 50% acetonitrile and 5% formic acid. Finally, samples were concentrated with the SpeedVac system up to 10 µl.

#### 2.5.5 Mass Spectrometry Analysis

The obtained peptides were analyzed with a liquid chromatography tandem mass spectrometric assay (LC-MS/MS) using an ion-trap time-of-flight (IT-TOF) instrument. An electrospray ion-trap time-of-flight (ESI-IT-TOF) mass spectrometer (Shimadzu Co) from the mass spectrometry facility from Universidad de São Paulo was used, according to a previously published protocol (29).

Protein sequence results were acquired in Mascot Generic Format (MGF) using Protein Post Rus Analyses (Shimadzu Co) and analyzed by Mascot Server version 2.4 or Peaks Studio version 7 using a personalized database for all *Leishmania* genus sequences from UniProt with the following parameters: 1 lost cleavage, mass tolerance 0.2 Da, carbamide-methyl-cysteine fixed modification, and variable of oxidized methionine.

The following parameters were taken into account to consider the protein sequence as positive: coverage percentage, number of peptides (minimum 2), and -10log *p*-value (must be equal to or higher than 50). Also, peptides from selected sequences were compared with theoretical peptides after digestion with trypsin using a Peptide Cutter tool (https://expasy.org/tools/peptidecutter) to confirm the sequences of the identified proteins.

#### 2.5.6 Bioinformatic Analysis

##### 2.5.6.1 Determination of Experimental and Theoretical Molecular Weight and Isoelectric Point

The experimental isoelectric points (pIs) and molecular weights (Mws) of identified spots were calculated using the Image Master Platinum program (GE HealthCare). Theoretical Mw and pI were calculated using the Compute pI/Mw bioinformatic tool (https://www.expasy.org/compuete_pi).

##### 2.5.6.2 Alignment of Protein Sequences by pBLAST

The protein sequences identified by mass spectrometry were analyzed by alignment of sequence using protein–protein Basic Local Alignment Search Tool (pBLAST). Sequences were compared with a protein database of the *Leishmania* genus, Trypanosomatidae family, *Homo sapiens*, and *Canis familiaris lupus* species.

##### 2.5.6.3 Protein Function Analysis

In order to assign a possible function to the identified proteins, they were analyzed by gene ontology (GEO) using the InterPro database (https://www.ebi.ac.uk/interpro/).

##### 2.5.6.4 Identification of Conserved Domains

Conserved domains of proteins were analyzed by UniProt (https://www.uniprot.org/database/DB-0214) and NCBI (https://www.ncbi.nlm.nih.gov/Structure/cdd/wrpsb.cgi) conserved domain databases. The second one uses a Reverse Position-Specific BLAST.

### 2.6 Statistical Analysis

Statistically significant differences among groups were evaluated by parametric (one or two-way ANOVA) or non-parametric (Kruskal–Wallis) tests and Dunnet’s, Bonferroni, or Dunn’s post-tests according to each case, using R studio and GraphPad Prism 5.0 software. The displayed results are representative of two independent experiments.

## 3 Results

### 3.1 TLA + Poly (I:C) + Montanide Formulation Enhances the Immune Response

**Figure 1:**
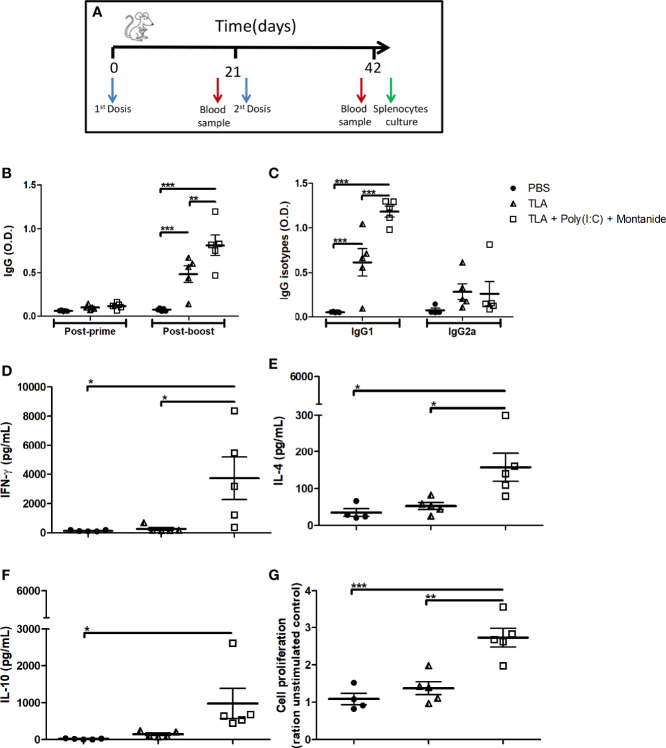
Humoral and cellular immune response in immunized mice. Vaccination scheme **(A)**. Anti-*Leishmania* IgG antibodies **(B)** using serum samples dilution (1:500) obtained 3 weeks after each immunization. Anti-*Leishmania* IgG1 and IgG2a **(C)** antibodies using serum samples dilution (1:1,000) obtained 3 weeks after boost. Results are presented as the mean ± standard error of mean (SEM) of optical density (O.D.) values from biological replicates obtained from 5 animals per groups. Cytokine levels of IFN-γ **(D)**, IL-4 **(E)**, and IL-10 **(F)** measured by enzyme-linked immunosorbent assay (ELISA) using supernatant of splenocytes stimulated with TLA for 72 h 3 weeks after boost; results are presented as the mean ± SEM of cytokine concentration (pg) from biological replicates obtained from 5 animals per group. Cell proliferation assay **(G)** determined by MTT on stimulated splenocytes with TLA for 72 h 3 weeks after boost; results are presented as the mean ± SEM of the cell proliferation index (ratio of unstimulated control) from biological replicates obtained from 5 animals per group. Asterisks indicate significant differences among groups: *p < 0.05; **p < 0.01; ***p < 0.001 by one-way **(C–G)** or two-way **(A, B)** ANOVA and Tukey’s or Bonferroni post-tests.

To evaluate whether TLA + Poly (I:C) + Montanide formulation induces a strong immune response, five BALB/c mice per group were immunized twice with a first-generation vaccine; TLA-specific IgGs were measured 21 days after each immunization; and IgG1/IgG2a, cytokine production, and cell proliferation were measured 21 days after the booster (**Figure 1A**). As it can be seen in **Figure 1B**, both TLA alone and TLA combined with adjuvants induced seroconversion after the second dose (*p* < 0.001). However, IgG antibodies were higher in the group that received TLA + Poly (I:C) + Montanide ISA 763 (*p* < 0.01). Both groups of animals produced high levels of IgG1 (*p* < 0.001) but not of IgG2a (**Figure 1C**).

The adjuvanted vaccine formulation induced higher levels of IFN-γ, IL-4, and cell proliferation than both control groups (**Figures 1D, E, G**), as well as higher levels of IL-10 than the PBS control group (**Figure 1F**).

### 3.2 TLA + Poly (I:C) + Montanide Ameliorates L. amazonensis Infection

**Figure 2:**
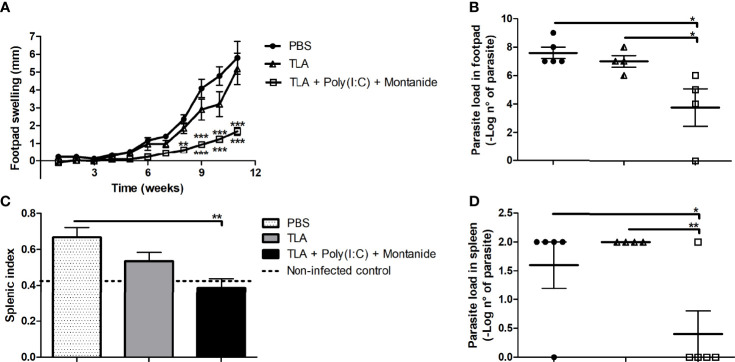
Protection analysis in immunized mice. Five animals per group were infected with 10,000 promastigotes of *L. amazonensis* by intradermic injection *via* the right footpad 14 days after the last boost. Footpad swelling (mm) was measured weekly until 11 weeks after infection; data are presented as the mean ± SEM **(A)**. Parasite load was determined by limiting dilution in the right footpad 11 weeks after infection; data are presented as the mean ± SEM of –log number of parasites **(B)**. Splenic index was calculated 11 weeks after infection; data are presented as the mean ± SEM **(C)**. Parasite load was determined by limiting dilution in the spleen 11 weeks after infection; data are presented as the mean ± SEM of –log number of parasites **(D)**. Asterisks represent differences between groups *p < 0.05; **p < 0.01; ***p < 0.001 by ANOVA **(A–C)** and Kruskal–Wallis **(D)** tests.

To evaluate protection against *L. amazonensis* infection, five animals per group received 3 doses of one formula subcutaneously in the interscapular region and were challenged on the right footpad 14 days after the last immunization with *L. amazonensis* promastigotes. The results shown in **Figure 2** demonstrate that mice immunized with TLA + Poly (I:C) + Montanide ISA 763 displayed a reduced *L. amazonensis* infection. Vaccinated mice with TLA + Poly (I:C) + Montanide ISA 763 showed a smaller lesion size than the PBS control group from week 8 (*p* < 0.01, asterisks above the line) as well as the TLA control group since week 9 (*p* < 0.001, asterisks under the line) (**Figure 2A**). These results were maintained until the end of the protocol at week 11 (*p* < 0.001). The adjuvanted vaccine formulation reduced parasite load on the footpad and the spleen compared with PBS and TLA control groups (**Figures 2B, D**). Mice immunized with TLA + Poly (I:C) + Montanide showed less splenic index than the PBS control group (**Figure 2C**), with similar values to non-infected mice. An increase of splenic index indicates splenomegaly, which is an indicator of inflammation due to the arrival of parasite to the spleen.

### 3.3 Identification of Four Leishmania Antigens by Immunoproteomic Analysis

**Figure 3:**
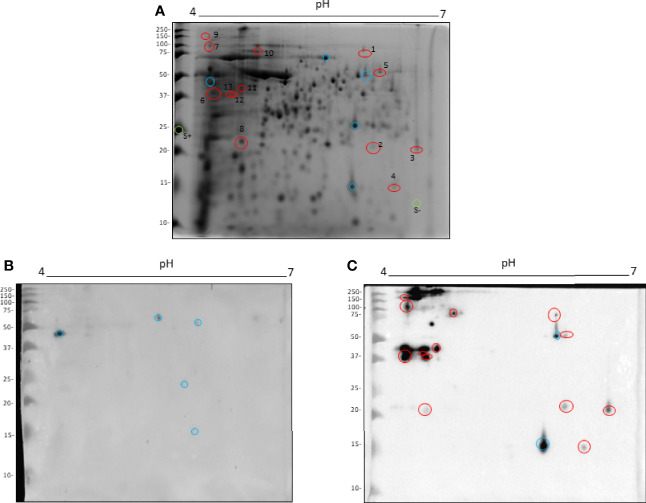
Immunoproteomic analysis by 2D Western blot. Total *L. amazonensis* proteins distributed in a 2D SDS-PAGE stained with Comassie Brillant Blue **(A)**. Membrane of 2D Western blot incubated with a pool of serum from the PBS control mice **(B)**. Membrane of 2D Western blot incubated with a pool of serum from mice immunized with TLA + Poly (I:C) + Montanide ISA 763 **(C)**. Blue circles represent spots recognized by the PBS control serum. Red circles represent spots analyzed by mass spectrometry. Green circles represent negative and positive controls of protein in gel.

Total proteins of *L. amazonensis* were separated in 2D electrophoresis gel according to their isoelectric point and molecular weight (**Figure 3A**). In order to evaluate the antigenicity of obtained spots, immunoblotting was carried out and the membranes were incubated with serum from mice receiving PBS (as an unspecified control) or TLA + Poly (I:C) + Montanide ISA 763 (**Figures 3B, C**). Western blot analysis showed that serum from the PBS control mice recognized 5 nonspecific proteins (shown as blue circles in **Figure 3B**).

Moreover, a large amount of proteins were recognized by serum from immunoprotected mice. These proteins were mainly distributed between 35 and 250 kDa with a pI between 4 and 5 (**Figure 3C**). Two spots were recognized by both sera, corresponding to a cross-reactivity (blue circles in **Figure 3C**); therefore, those were excluded in the protein sequence identification.

From the observed proteins, 13 spots were excised from gel (red circles), with a positive and a negative control (green circles, S+ and S-) to be analyzed by mass spectrometry.

As seen in **Table 1**, the amino acid sequences from four spots (3, 6, 7, and 11) were identified by mass spectrometry and considered positive according to the inclusion criteria detailed in the Materials and Methods section.

**Table 1:** Characteristics of the identified immunodominant proteins.

Spot	Exp. pI	Theor. pI	Exp. Mw	Theor. Mw	Name of the protein (*Leishmania* species)	Accession no. (UniProt/NCBI)
**3**	7.002	6.89	21	21	**Cytosolic tryparedoxin peroxidase** (*L. amazonensis*)	Q4VKK8 AAX47428.1
**6**	4.25	5.28	39	38	**Uncharacterized protein** (*L. mexicana*)	E9AL77 XP_003872211.1
**7**	4.199	4.22	105	72	**Kinetoplast-associated protein-like protein** (*L. infantum*)	A4I2L3 XP_001466296.1
**11**	4.627	6.51	44	43.6	**Putative heat-shock protein DNAJ** (*L. major*)	E9ACW0 XP_003722052.1

### 3.4 Analysis of Families, Domains, Functions, and Conservation of Identified Proteins

Families and domains of identified proteins were analyzed by the UniProt database, whereas their function was analyzed by gene ontology analysis using the InterPro database. Using the BLASTp bioinformatic tool, protein sequences were compared with the database of the *Leishmania* genus (Taxid: 5658), Trypanasomatid family (Taxid: 5654), *Homo sapiens* (Taxid: 9606), and *Canis lupus familiaris* (Taxid: 9615) (**Supplementary Files**).

Firstly, the cytosolic tryparedoxin peroxidase (L. amazonensis) protein contains 199 amino acids, with a thioredoxin domain at the 6–165 position. This domain mediates its participation in redox reactions, usually *via* reversible oxidation of a cysteine residue leading to a cysteine-sulfenic acid that can either be stabilized or react with an unmodified cysteine residue and form a stable but reversible disulfide bond. When a GEO analysis was carried out, antioxidant, peroxiredoxin, and oxidoreductase activities were found.

Secondly, according to the UniProt analysis, the uncharacterized protein (*L. mexicana*) contains a coiled-coil domain at the 88–115 position. A coiled coil is a type of secondary structure composed of two or more alpha helices that entwine to form a cable structure. In proteins, the helical cables perform a mechanical role in forming stiff bundles of fibers. When a GEO analysis was carried out on this protein, no GEO terms were found. Using the NCBI conserved domains database, it was observed that this protein showed a non-specific hit. This hit was an Mpr1p, Pad1p N-terminal (MPN) domain without catalytic isopeptidase activity found in the eukaryotic translation initiation factor 2 (eIF2) subunit h (eIF2h), which is related to protein synthesis.

Thirdly, according to UniProt, no domains were identified in the kinetoplast-associated protein-like protein (KAP) (*L. infantum*); as a result, the entirety of its 660-amino-acid-long sequence revealed a disordered region. No GEO terms were found either. However, using the NCBI database, two specific hits were found: a trichohyalin–plectin–homology (TPH) domain and a TolA protein. The TPH domain has been associated with mitochondrial movement, whereas the TolA protein is part of the Tol–Pal complex that is required for maintaining outer-membrane integrity.

Finally, according to the NCBI and UniProt analysis, the putative heat-shock protein (HSP) DNAJ (*L. major*) showed a DNAJ domain at the 6–70 position and a molecular chaperone with the C-terminal Zn finger domain at the 120–205 position. The GEO results showed that this protein participates in the biological process of protein folding and heat response. In addition, this protein has different molecular functions related among them: ATP binding, HSP70 protein binding, metal ion binding, and unfolded protein binding.

According to the BLASTp bioinformatic tool, the four identified proteins showed a highly conserved level (up to 99% of identity) among *Leishmania* species, which causes different clinical manifestations. Also, all these proteins, except the uncharacterized protein from *L. mexicana*, are highly conserved among species belonging to the Trypanosomatid family, which causes human disease, such as Chagas and African trypanosomiasis. Furthermore, all these proteins showed a low level of protein conservation when compared to all proteins of the human and dog database. In those cases, the percentage of identity was lower than 60% with no sequence identity for the uncharacterized protein from *L. mexicana* (**Supplemetary File**).

## 4 Discussion

Leishmaniasis is a parasitic disease for which there is no available vaccine for human use (7). As a result, continuous research is needed in order to develop a vaccine. In this study, a combination of two adjuvants with different action mechanisms was proposed to enhance vaccine protection against *L. amazonensis*. Hence, TLA was formulated with Poly (I:C) and Montanide ISA 763, and its immune response and protective effect were evaluated. The synergistic priming effect on the immune response to the antigen given by the combination of both adjuvants has been suggested by other authors in a therapeutic cancer vaccine (30).

In the current study, the formulation containing TLA, Poly (I:C), and Montanide ISA 763 induced an increased humoral and cellular immune response, characterized by high levels of total IgG and IgG1 antibodies, IFN-γ, IL-4, and IL-10. Despite increases in all cytokines given by the immunization with this formula, IFN-γ levels were higher than the others. Unfortunately, with this approach, it is still unclear which immune cell subset (CD4 T, CD8, or B cells) is responsible for IFN-γ production. However, more studies concerning T-cell-dependent immune responses before and after infection would be necessary to better understand underlying mechanisms given by the evaluated vaccine formula and how they are modulated by the ongoing *L. amazonensis* infection. As *L. amazonensis* may infect APCs and skew T-cell priming, the possibility that *L. amazonensis* infection may modulate the vaccine-induced T-cell response should not be discarded (31).

The role of antibodies on *Leishmania* infection is unclear. Some authors suggest that increasing antibody titers after infection could help the parasite’s entry into the host cells and the development of the disease (32). In contrast, other authors suggest that disease outcome depends on different factors, such as the infective dose, the quantity of opsonized parasites, the interaction between IgG, and the different Fc-γ receptors, among others (33). Moreover, Rostamian and colleagues observed that a protective adjuvated recombinant protein increased IgG1 antibodies after immunization and decreased them after challenge (34). As a result, more detailed studies are needed to evaluate the humoral immune response after infection to understand the observed results.

Even though TLA + Poly (I:C) + Montanide induced a mixed Th1:Th2:Treg immune response, it was able to improve BALB/c mice from infection by *L. amazonensis*. This achievement was reflected in their smaller lesion size, lower splenic index, and lower parasite load on the footpad and spleen than the control group. Similar results were found by Shakri and collaborators using a vaccine formulated with the recombinant *L. major* stress-inducible protein 1 (LmSTI1) and Montanide ISA 50 against infection by *L. major* (35). They showed that even though the vaccine enhanced the IFN-γ, IL-4, IL-10, and IL-17 cytokines, this also decreased lesion size and parasite load. Additionally, it has been observed that asymptomatic patients show a low parasite load, due to the increase in pro-inflammatory cytokines controlled by the IL-10 production (36). Our results demonstrate the importance of a balance between IFN-y and IL-10 production in order to control the disease without causing tissular damage.

Therefore, stimulated splenocytes from mice immunized with TLA and adjuvants exhibited a significant increase in cell proliferation. Although cell proliferation of splenocytes does not have a direct correlation with effector function of T lymphocytes, this is broadly employed to evaluate the immunological characteristics elicited by antigens in vaccines (37).

The scheme of immunization and site of infection could change the outcome of a given vaccine. Aligned with our data, Mehravaran and colleagues performed an *L. amazonensis* challenge 14 days after the last booster, which obtained good results (38). However, Salari et al. and Lage et al. demonstrated that their vaccines also displayed protection from *L. amazonensis* challenge 3–4 weeks after the last booster, indicating that different *L. amazonensis* challenge protocols may be valuable and adaptable (39, 40). Moreover, it has been demonstrated that the site of the *Leishmania* infection determines disease severity and immune response (41).

In the present study, a serological-proteomic analysis was made by two-dimensional Western blot, where total *L. amazonensis* proteins were separated in 2D gel and incubated with a pool of sera from immunoprotected BALB/c mice. A similar approach was successful in order to identify antigens used for experimental vaccines (42, 43). Here, four immunodominant proteins were identified, which are possible candidates to produce new-generation vaccines based on one protein. The identified proteins were as follows: cytosolic tryparedoxin peroxidase from *L. amazonensis*, a non-characterized protein from *L. mexicana*, a KAP from *L. infantum*, and a putative HSP DNAJ from *L. major*.

As it can be seen in **Table 1**, identified proteins were not from *L. amazonensis* in all the cases, despite being the species used for the analysis. The reason for this could be that not all protein sequences of *L. amazonensis* are in the database. As a result, all *Leishmania* spp. databases were used for the analysis. However, some differences between theoretical and experimental pI and Mw were observed, mainly in the KAP of *L. infantum*. This could be due to post-translation modifications and protein processing observed in *Leishmania* spp., even in the KAP of *Leishmania* (44, 45).

One of the identified proteins was the cytosolic tryparedoxin peroxidase. It belongs to the 2-cysteine peroxiredoxin family and has been characterized on *Leishmania* genus and other trypanosomatids. This protein is a virulence factor that participates against the oxidative stress as an antioxidant enzyme *via* metabolism of hydrogen peroxide to water. For that, it has an important role in the survival of parasites inside the target cells, as well as in drug resistance (46, 47). Moreover, it has been demonstrated that this protein is expressed in the promastigote and amastigote forms (48). In accordance with previous reports, we observed that cytosolic tryparedoxin peroxidase is highly conserved in trypanosomatids protozoa (49). It has been evidenced that this protein elicits IL-4 and IL-10 expression, and it also suppresses IL-12 and IFN-γ expression in blood-derived monocytes from patients with visceral leishmaniasis. Hence, this protein acts as an immunosuppressant, promoting disease progression (50). Nevertheless, this protein has not been used as an antigen for vaccine development against leishmaniasis.

Furthermore, a KAP was recognized by sera from immunized mice. There are different types of KAPs, and their function and location have been studied on different kinetoplastid protozoa, but not in *Leishmania* spp (51–53). Its main function is related to organizing the kDNA network, guaranteeing the packaging and segregation of mitochondrial DNA (mtDNA) (54). However, it has been demonstrated that KAP6 depletion in *T. brucei* parasites stopped its cell growth (52), although it was not observed on either KAP3 knockout *T. cruzi* parasites or KAP2 and KAP3 double-knockout *Crithidia fasciculata* (51, 53).

Finally, the identified HSP DNAJ, just like the other members of the HSP family, is a highly conserved molecular chaperone. It has been demonstrated that it has an important function in the life cycle and survival of parasites inside mammalian host cells (55). Several virulence factors, including HSP, have been used as antigens to develop vaccines against leishmaniasis (56). In particular, HSP has been used as a vaccine antigen against other infectious diseases and also against cancer. The HSP antigen has been used as peptide, protein, and DNA vaccines (57).

Some authors suggest that antigens that promote a susceptibility-inducing immune response during early infection could be protective when combined with Th1-promoting adjuvants in prophylactic vaccines (58). These data support the idea that virulence factors such as tryparedoxin peroxidase and HSP can be promising candidates to develop a one-protein vaccine against leishmaniasis. The proteins identified by immunoblot may not precisely induce a cellular immune response as the IgG levels must be identified and T-cell peptide analysis must be carried out to fulfill this task. However, the peptide nature and the IgG-switched humoral immune response against these antigens may reflect a conventional germinal center reaction where T–B cell cooperation and T-cell priming have occurred. The T-cell-specific immunodominance from these proteins would shed light on *L. amazonensis* biology to develop a more effective vaccine. The study of the T-cell epitopes of these peptides was not included in the scope of this study.

Additionally, a vaccine antigen against *Leishmania* should be immunogenic, conserved among the *Leishmania* species, and phylogenetically divergent to any human or canine protein (according to the host where the vaccine will be used) to avoid autoimmunity (59). Here, it can be demonstrated that identified proteins showed a low phylogenetic conservation when they were compared to the human and canine protein databases. In addition, these proteins manifested a high conservation compared to the *Leishmania* genus and even the Trypanosomatid family protein databases.

In conclusion, all these data suggest that TLA and Poly (I:C) emulsified with Montanide ISA 763 could be a good candidate for a first-generation vaccine against cutaneous leishmaniasis caused by *L. amazonensis*. Moreover, the four identified proteins from the first-generation vaccine could be protective immunodominant antigens to be considered for vaccine development against leishmaniasis disease.

## Data Availability Statement

The original contributions presented in the study are included in the article/**Supplementary Material**. Further inquiries can be directed to the corresponding author.

## Ethics Statement

The animal study was reviewed and approved by the Institutional Animal Care and Use Committee of the Facultad de Ciencias Médicas, Universidad Nacional de Cuyo (protocol approval n°: 80/2016).

## Author Contributions

Study conception and design: MG, AF, and DC. Data collection: MG, JM-O, MD, JV, DP, EL, MS, and FB. Analysis and interpretation of results: MG, JM-O, AF, and DC. Draft manuscript preparation: MG, JM-O, AF, and DC. All authors contributed to the article and approved the submitted version.

## Funding

This work was supported by Universidad Nacional de Cuyo (UNCuyo SIIP 06/J499; UNCuyo SIIP J082/2019), Consejo Nacional de Investigaciones Científicas y Técnicas (CONICET) (PIP 11220150100210Co 2015-2017 and PIP 0081-2015), Agencia Nacional de Promoción Científica y Tecnológica (AGENCIA) (PICT 2018-01511, PICT-2015-0271, PICT 2015-1052, PICT 2018-2642, and PICT 2019-01762), Universidad del Aconcagua (CIUDA 2021). Instituto Nacional de Ciência e Tecnologia em Vacinas (INCTV)/CNPq (grant nº 465293/2014-0), and Rede Mineira de Biomoléculas (FAPEMIG grant nº red001214). Authors received research and students fellowships from Consejo Nacional de Investigaciones Científicas y Técnicas (CONICET), Conselho Nacional de Desenvolvimento Científico e Tecnológico (CNPq, Brazil) and Coordenação de Aperfeiçoamento de Pessoal de Nível Superior (CAPES, Brazil), respectively.

## Conflict of Interest

The authors declare that the research was conducted in the absence of any commercial or financial relationships that could be construed as a potential conflict of interest.

## Publisher’s Note

All claims expressed in this article are solely those of the authors and do not necessarily represent those of their affiliated organizations, or those of the publisher, the editors and the reviewers. Any product that may be evaluated in this article, or claim that may be made by its manufacturer, is not guaranteed or endorsed by the publisher.

## References

[1] WHO. Control of the Leishmaniases. Geneva: WHO (2010). WHO.

[2] Antinori S Schifanella L Corbellino M. Leishmaniasis: New Insights From an Old and Neglected Disease. Eur J Clin Microbiol Infect Dis (2012) 31(2):109–18. doi: 10.1007/s10096-011-1276-0 21533874

[3] Jara M Adaui V Valencia BM Martinez D Alba M Castrillon C. Real-Time PCR Assay for Detection and Quantification of Leishmania (Viannia) Organisms in Skin and Mucosal Lesions: Exploratory Study of Parasite Load and Clinical Parameters. J Clin Microbiol (2013) 51(6):1826–33. doi: 10.1128/JCM.00208-13 PMC3716068 23554201

[4] Akilov OE Khachemoune A Hasan T. Clinical Manifestations and Classification of Old World Cutaneous Leishmaniasis. Int J Dermatol (2007) 46(2):132–42. doi: 10.1111/j.1365-4632.2007.03154.x 17269962

[5] Germano MJ Salomón MC Neira G Lozano E Mackern-Oberti JP Cargnelutti DE. Leishmaniasis in the Argentine Republic: Temporal and Geographical Distribution From 2013 to 2017. Asian Pac J Trop Med (2019) 12:300–5. doi: 10.4103/1995-7645.262073

[6] Christensen SM Belew AT El-Sayed NM Tafuri WL Silveira FT Mosser DM. Host and Parasite Responses in Human Diffuse Cutaneous Leishmaniasis Caused by L. amazonensis PloS Negl Trop Dis (2019) 13(3):e0007152. doi: 10.1371/journal.pntd.0007152 30845223 PMC6405045

[7] Mohebali M Nadim A Khamesipour A. An Overview of Leishmanization Experience: A Successful Control Measure and a Tool to Evaluate Candidate Vaccines. Acta Trop (2019) 200:105173. doi: 10.1016/j.actatropica.2019.105173 31525323

[8] Velez R Gallego M. Commercially Approved Vaccines for Canine Leishmaniosis: A Review of Available Data on Their Safety and Efficacy. Trop Med Int Health (2020) 25(5):540–57. doi: 10.1111/tmi.13382 32034985

[9] Srivastava S Shankar P Mishra J Singh S. Possibilities and Challenges for Developing a Successful Vaccine for Leishmaniasis. Parasit Vectors (2016) 9(1):277. doi: 10.1186/s13071-016-1553-y 27175732 PMC4866332

[10] Rossi M Fasel N. How to Master the Host Immune System? Leishmania Parasites Have the Solutions! Int Immunol (2018) 30(3):103–11. doi: 10.1093/intimm/dxx075 PMC5892169 29294040

[11] Akhoundi M Kuhls K Cannet A Votypka J Marty P Delaunay P. A Historical Overview of the Classification, Evolution, and Dispersion of Leishmania Parasites and Sandflies. PloS Negl Trop Dis (2016) 10(3):e0004349. doi: 10.1371/journal.pntd.0004349 26937644 PMC4777430

[12] Almeida APMM Machado LFM Doro D Nascimento FC Damasceno L Gazzinelli RT. New Vaccine Formulations Containing a Modified Version of the Amastigote 2 Antigen and the Non-Virulent Trypanosoma Cruzi CL-14 Strain Are Highly Antigenic and Protective Against Leishmania Infantum Challenge. Front Immunol (2018) 9:465. doi: 10.3389/fimmu.2018.00465 29599776 PMC5863692

[13] Thompson EA Lore K. Non-Human Primates as a Model for Understanding the Mechanism of Action of Toll-Like Receptor-Based Vaccine Adjuvants. Curr Opin Immunol (2017) 47:1–7. doi: 10.1016/j.coi.2017.06.006 28715767

[14] Martins KA Bavari S Salazar AM. Vaccine Adjuvant Uses of Poly-IC and Derivatives. Expert Rev Vaccines (2015) 14(3):447–59. doi: 10.1586/14760584.2015.966085 25308798

[15] Salem ML El-Naggar SA Kadima A Gillanders WE Cole DJ. The Adjuvant Effects of the Toll-Like Receptor 3 Ligand Polyinosinic-Cytidylic Acid Poly (I:C) on Antigen-Specific CD8+ T Cell Responses are Partially Dependent on NK Cells With the Induction of a Beneficial Cytokine Milieu. Vaccine (2006) 24(24):5119–32. doi: 10.1016/j.vaccine.2006.04.010 16704888

[16] Germano MJ Lozano ES Sanchez MV Bruna FA Garcia-Bustos MF Sosa Lochedino AL. Evaluation of Different Total Leishmania Amazonensis Antigens for the Development of a First-Generation Vaccine Formulated With a Toll-Like Receptor-3 Agonist to Prevent Cutaneous Leishmaniasis. Mem Inst Oswaldo Cruz (2020) 115:e200067. doi: 10.1590/0074-02760200067 32667458 PMC7357544

[17] Sanchez MV Elicabe RJ Di Genaro MS Germano MJ Gea S Garcia Bustos MF. Total Leishmania Antigens With Poly(I:C) Induce Th1 Protective Response. Parasite Immunol (2017) 39(11):e12491. doi: 10.1111/pim.12491 28901553

[18] Hafner AM Corthesy B. Merkle HP. Particulate Formulations for the Delivery of Poly(I:C) as Vaccine Adjuvant. Adv Drug Delivery Rev (2013) 65(10):1386–99. doi: 10.1016/j.addr.2013.05.013 23751781

[19] Aucouturier J Dupuis L Ganne V. Adjuvants Designed for Veterinary and Human Vaccines. Vaccine (2001) 19(17-19):2666–72. doi: 10.1016/s0264-410x(00)00498-9 11257407

[20] Cargnelutti DE Salomon MC Celedon V Garcia Bustos MF Morea G Cuello-Carrion FD. Immunization With Antigenic Extracts of Leishmania Associated With Montanide ISA 763 Adjuvant Induces Partial Protection in BALB/c Mice Against Leishmania (Leishmania) Amazonensis Infection. J Microbiol Immunol Infect (2016) 49(1):24–32. doi: 10.1016/j.jmii.2014.01.006 24662018

[21] Mutiso JM Macharia JC Kariuki TM Gicheru MM. Montanide ISA 720 Is More Effective Than BCG as an Adjuvant for Leishmania Killed Vaccine in BALB/c Mice. Int J Integ Biol (2009) 7(2):107–16.

[22] Mutiso JM Macharia JC Mutisya RM Taracha E. Subcutaneous Immunization Against Leishmania Major - Infection in Mice: Efficacy of Formalin-Killed Promastigotes Combined With Adjuvants. Rev Inst Med Trop Sao Paulo (2010) 52(2):95–100. doi: 10.1590/s0036-46652010000200006 20464130

[23] Skwarczynski M Toth I. Peptide-Based Synthetic Vaccines. Chem Sci (2016) 7(2):842–54. doi: 10.1039/c5sc03892h PMC5529997 28791117

[24] Costa MM Andrade HM Bartholomeu DC Freitas LM Pires SF Chapeaurouge AD. Analysis of Leishmania Chagasi by 2-D Difference Gel Electrophoresis (2-D DIGE) and Immunoproteomic: Identification of Novel Candidate Antigens for Diagnostic Tests and Vaccine. J Proteome Res (2011) 10(5):2172–84. doi: 10.1021/pr101286y 21355625

[25] Falisse-Poirrier N Ruelle V ElMoualij B Zorzi D Pierard O Heinen E. Advances in Immunoproteomics for Serological Characterization of Microbial Antigens. J Microbiol Methods (2006) 67(3):593–6. doi: 10.1016/j.mimet.2006.05.002 16822569

[26] Dennehy R McClean S. Immunoproteomics: The Key to Discovery of New Vaccine Antigens Against Bacterial Respiratory Infections. Curr Protein Pept Sci (2012) 13(8):807–15. doi: 10.2174/138920312804871184 PMC3594738 23305366

[27] Mosmann T. Rapid Colorimetric Assay for Cellular Growth and Survival: Application to Proliferation and Cytotoxicity Assays. J Immunol Methods (1983) 65(1-2):55–63. doi: 10.1016/0022-1759(83)90303-4 6606682

[28] Bogdan C Debus A Sebald H Rai B Schäfer J Obermeyer S. Experimental Cutaneous Leishmaniasis: Mouse Models for Resolution of Inflammation Versus Chronicity of Disease. Methods Mol Biol (2019) 1971:315–49. doi: 10.1007/978-1-4939-9210-2_18 30980313

[29] Duarte MC Pimenta DC Menezes-Souza D Magalhaes RD Diniz JL Costa LE. Proteins Selected in Leishmania (Viannia) Braziliensis by an Immunoproteomic Approach With Potential Serodiagnosis Applications for Tegumentary Leishmaniasis. Clin Vaccine Immunol (2015) 22(11):1187–96. doi: 10.1128/CVI.00465-15 PMC4622107 26376929

[30] Pavlick A Blazquez AB Meseck M Lattanzi M Ott PA Marron TU. Combined Vaccination With NY-ESO-1 Protein, Poly-ICLC, and Montanide Improves Humoral and Cellular Immune Responses in Patients With High-Risk Melanoma. Cancer Immunol Res (2020) 8(1):70–80. doi: 10.1158/2326-6066.CIR-19-0545 31699709 PMC6946846

[31] Xin L Li Y Soong L. Role of Interleukin-1beta in Activating the CD11c(high) CD45RB- Dendritic Cell Subset and Priming Leishmania Amazonensis-Specific CD4+ T Cells In Vitro and *In Vivo* . Infect Immun (2007) 75(10):5018–26. doi: 10.1128/IAI.00499-07 PMC2044509 17682041

[32] de Lima C Magalhães AS Costa R Barreto CC Machado P Carvalho EM. High Anti-Leishmania IgG Antibody Levels Are Associated With Severity of Mucosal Leishmaniasis. Front Cell Infect Microbiol (2021) 11:652956. doi: 10.3389/fcimb.2021.652956 33898330 PMC8063102

[33] Gardinassi LG de Miranda Santos IKF. Comment on “Regulation of Immunity During Visceral Leishmania Infection” and Further Discussions About the Role of Antibodies in Infections With Leishmania. Parasit Vectors (2016) 9(1):1–4. doi: 10.1186/s13071-016-1669-0 27387545 PMC4936235

[34] Rostamian M Niknam HM. Role of Higher Levels of Post-Challenge Antibodies in Protective Vaccination Against Leishmania Tropica Infection of BALB/c Mice. Asian Pac J Trop Biomed (2020) 10(12):532–9. doi: 10.4103/2221-1691.297052

[35] Shokri M Roohvand F Alimohammadian MH Ebrahimirad M Ajdary S. Comparing Montanide ISA 720 and 50-V2 Adjuvants Formulated With LmSTI1 Protein of Leishmania Major Indicated the Potential Cytokine Patterns for Induction of Protective Immune Responses in BALB/c Mice. Mol Immunol (2016) 76:108–15. doi: 10.1016/j.molimm.2016.06.010 27428863

[36] Iborra S Solana JC Requena JM Soto M. Vaccine Candidates Against Leishmania Under Current Research. Expert Rev Vaccines (2018) 17(4):323–34. doi: 10.1080/14760584.2018.1459191 29589966

[37] Lagerqvist N Naslund J Lundkvist A Bouloy M Ahlm C Bucht G. Characterisation of Immune Responses and Protective Efficacy in Mice After Immunisation With Rift Valley Fever Virus cDNA Constructs. Virol J (2009) 6:6. doi: 10.1186/1743-422X-6-6 19149901 PMC2637244

[38] Mehravaran A Nasab MR Mirahmadi H Sharifi I Alijani E Nikpoor AR. Protection Induced by Leishmania Major Antigens and the Imiquimod Adjuvant Encapsulated on Liposomes in Experimental Cutaneous Leishmaniasis. Infect Genet Evol (2019) 70:27–35. doi: 10.1016/j.meegid.2019.01.005 30738195

[39] Salari S Sharifi I Keyhani AR Almani PGN. Evaluation of a New Live Recombinant Vaccine Against Cutaneous Leishmaniasis in BALB/c Mice. Parasit Vectors (2020) 13(1):1–16. doi: 10.1186/s13071-020-04289-7 32787908 PMC7425157

[40] Lage DP Ribeiro PA Dias DS Mendonça DV Ramos FF Carvalho LM. A Candidate Vaccine for Human Visceral Leishmaniasis Based on a Specific T Cell Epitope-Containing Chimeric Protein Protects Mice Against Leishmania Infantum Infection. NPJ Vaccines (2020) 5(1):1–13. doi: 10.1038/s41541-020-00224-0 32821440 PMC7426426

[41] Baldwin TM Elso C Curtis J Buckingham L Handman E. The Site of Leishmania Major Infection Determines Disease Severity and Immune Responses. Infect Immun (2003) 71(12):6830–4. doi: 10.1128/IAI.71.12.6830-6834.2003 PMC308923 14638769

[42] Coelho VT Oliveira JS Valadares DG Chavez-Fumagalli MA Duarte MC Lage PS. Identification of Proteins in Promastigote and Amastigote-Like Leishmania Using an Immunoproteomic Approach. PloS Negl Trop Dis (2012) 6(1):e1430. doi: 10.1371/journal.pntd.0001430 22272364 PMC3260309

[43] Martins VT Chavez-Fumagalli MA Lage DP Duarte MC Garde E Costa LE. Antigenicity, Immunogenicity and Protective Efficacy of Three Proteins Expressed in the Promastigote and Amastigote Stages of Leishmania Infantum Against Visceral Leishmaniasis. PloS One (2015) 10(9):e0137683. doi: 10.1371/journal.pone.0137683 26367128 PMC4569552

[44] Brotherton MC Racine G Foucher AL Drummelsmith J Papadopoulou B Ouellette M. Analysis of Stage-Specific Expression of Basic Proteins in Leishmania Infantum. J Proteome Res (2010) 9(8):3842–53. doi: 10.1021/pr100048m 20583757

[45] Rosenzweig D Smith D Myler PJ Olafson RW Zilberstein D. Post-Translational Modification of Cellular Proteins During Leishmania Donovani Differentiation. Proteomics (2008) 8(9):1843–50. doi: 10.1002/pmic.200701043 18398879

[46] Andrade JM Murta SM. Functional Analysis of Cytosolic Tryparedoxin Peroxidase in Antimony-Resistant and -Susceptible Leishmania Braziliensis and Leishmania Infantum Lines. Parasit Vectors (2014) 7:406. doi: 10.1186/1756-3305-7-406 25174795 PMC4261743

[47] Iyer JP Kaprakkaden A Choudhary ML Shaha C. Crucial Role of Cytosolic Tryparedoxin Peroxidase in Leishmania Donovani Survival, Drug Response and Virulence. Mol Microbiol (2008) 68(2):372–91. doi: 10.1111/j.1365-2958.2008.06154.x 18312262

[48] Teixeira PC Velasquez LG Lepique AP de Rezende E Bonatto JM Barcinski MA. Regulation of Leishmania (L.) Amazonensis Protein Expression by Host T Cell Dependent Responses: Differential Expression of Oligopeptidase B, Tryparedoxin Peroxidase and HSP70 Isoforms in Amastigotes Isolated From BALB/c and BALB/c Nude Mice. PloS Negl Trop Dis (2015) 9(2):e0003411. doi: 10.1371/journal.pntd.0003411 25692783 PMC4333223

[49] Suman SS Equbal A Zaidi A Ansari MY Singh KP Singh K. Up-Regulation of Cytosolic Tryparedoxin in Amp B Resistant Isolates of Leishmania Donovani and its Interaction With Cytosolic Tryparedoxin Peroxidase. Biochimie (2016) 121:312–25. doi: 10.1016/j.biochi.2015.12.017 26743980

[50] Suman SS Amit A Singh KP Gupta P Equbal A Kumari A. Cytosolic Tryparedoxin of Leishmania Donovani Modulates Host Immune Response in Visceral Leishmaniasis. Cytokine (2018) 108:1–8. doi: 10.1016/j.cyto.2018.03.010 29554571

[51] de Souza FS Rampazzo Rde C Manhaes L Soares MJ Cavalcanti DP Krieger MA. Knockout of the Gene Encoding the Kinetoplast-Associated Protein 3 (KAP3) in Trypanosoma Cruzi: Effect on Kinetoplast Organization, Cell Proliferation and Differentiation. Mol Biochem Parasitol (2010) 172(2):90–8. doi: 10.1016/j.molbiopara.2010.03.014 20363262

[52] Wang J Pappas-Brown V Englund PT Jensen RE. TbKAP6, a Mitochondrial HMG Box-Containing Protein in Trypanosoma Brucei, is the First Trypanosomatid Kinetoplast-Associated Protein Essential for Kinetoplast DNA Replication and Maintenance. Eukaryot Cell (2014) 13(7):919–32. doi: 10.1128/EC.00260-13 PMC4135736 24879122

[53] Avliyakulov NK Lukes J Ray DS. Mitochondrial Histone-Like DNA-Binding Proteins are Essential for Normal Cell Growth and Mitochondrial Function in Crithidia Fasciculata. Eukaryot Cell (2004) 3(2):518–26. doi: 10.1128/EC.3.2.518-526.2004 PMC387644 15075280

[54] de Souza SSA Catta-Preta CM Alves JMP Cavalcanti DP Teixeira MMG Camargo EP. Expanded Repertoire of Kinetoplast Associated Proteins and Unique Mitochondrial DNA Arrangement of Symbiont-Bearing Trypanosomatids. PloS One (2017) 12(11):e0187516. doi: 10.1371/journal.pone.0187516 29131838 PMC5683618

[55] Kröber-Boncardo C GJ Clos J. Heat Shock Proteins. In: Leishmania Parasites. Switzerland:Springer. (2020). p. 1–20. Springer, editor.

[56] Sundar S Singh B. Identifying Vaccine Targets for Anti-Leishmanial Vaccine Development. Expert Rev Vaccines (2014) 13(4):489–505. doi: 10.1586/14760584.2014.894467 24606556 PMC4040457

[57] Bolhassani A Rafati S. Heat-Shock Proteins as Powerful Weapons in Vaccine Development. Expert Rev Vaccines (2008) 7(8):1185–99. doi: 10.1586/14760584.7.8.1185 18844593

[58] de Mendonca SC Cysne-Finkelstein L Matos DC. Kinetoplastid Membrane Protein-11 as a Vaccine Candidate and a Virulence Factor in Leishmania. Front Immunol (2015) 6:524. doi: 10.3389/fimmu.2015.00524 26528287 PMC4602152

[59] Cunningham AL Garcon N Leo O Friedland LR Strugnell R Laupeze B. Vaccine Development: From Concept to Early Clinical Testing. Vaccine (2016) 34(52):6655–64. doi: 10.1016/j.vaccine.2016.10.016 27769596

