# Supplementary material for: Identification of Immunodominant Antigens From a First-Generation Vaccine Against Cutaneous Leishmaniasis
Source: Front Immunol. 2022 May 12;13:825007. doi: 10.3389/fimmu.2022.825007 (PMC9133320; doi:10.3389/fimmu.2022.825007)
Supplement: Supplementary file 1 [file Table_1.docx]

BLASTp analysis made on identified proteins

| **Cytosolic tryparedoxin peroxidase** | | | | | | |
| --- | --- | --- | --- | --- | --- | --- |
| **Name** | **Max score** | **Total score** | **Query cover** | **E value** | **Per. Ident.** | **Accession** |
| Cytosolic tryparedoxin peroxidase (*L. amazonesis*) | 416 | 416 | 100% | 3,00E-150 | 100% | AAX47426.1 |
| Cytoplasmatic tryparedoxin peroxidase (*L. donovani*) | 403 | 403 | 100% | 3,00E-145 | 96.98% | ABP68405.1 |
| Tryparedoxin peroxidase (*L. mexicana* MHOM/GT/2001/U1103) | 397 | 397 | 100% | 5,00E-143 | 97.47% | XP_003873633.1 |
| Tryparedoxin peroxidase (*L. infantum*JPCM5) | 380 | 380 | 100% | 4,00E-136 | 89.95% | XP_001464446.1 |
| Peroxidoxin (*L. major*) | 375 | 375 | 100% | 3,00E-134 | 88.44% | AAC79432.1 |
| Tryparedoxin peroxidase (*L. major* strain Friedlin) | 375 | 375 | 100% | 3,00E-134 | 88.44% | XP_001682022.1 |
| Peroxidoxin 2 (*L. tropica*) | 372 | 372 | 100% | 6,00E-133 | 87.44% | AAZ23601.1 |
| Peroxidoxin 2 (*L. aethiopica*) | 371 | 371 | 100% | 2,00E-132 | 87.44% | AAZ23602.1 |
| Trypoaredoxin peroxidase 2 (*L. guyanensis*) | 352 | 352 | 100% | 3,00E-125 | 81.91% | AAV31765.1 |
| Trypoaredoxin peroxidase (*L. panamensis*) | 350 | 350 | 100% | 2,00E-124 | 81.41% | XP_010697482.1 |
| Trypoaredoxin peroxidase (*L. braziliensis* MHOM/BR/75/M2904) | 346 | 346 | 100% | 1,00E-122 | 80.40% | XP_001563558.1 |
|  |  |  |  |  |  |  |
| **Name** | **Max score** | **Total score** | **Query cover** | **E value** | **Per. Ident.** | **Accession** |
| Tryparedoxin peroxidase (*Trypanosoma brucei gambiense* DAL972) | 303 | 303 | 98% | 2,00E-105 | 71.57% | XP_011776500.1 |
| Tryparedoxin peroxidase (*T. vivax* Y486) | 302 | 302 | 98% | 5,00E-105 | 71.07% | CCC50392.1 |
| Tryparedoxin peroxidase (*T. cruzi cruzi*) | 298 | 298 | 98% | 2,00E-103 | 69.9% | PBJ78285.1 |
|  |  |  |  |  |  |  |
| **Name** | **Max score** | **Total score** | **Query cover** | **E value** | **Per. Ident.** | **Accession** |
| Human peroxiredoxin-1 C83S mutant (*H. sapiens*) | 258 | 258 | 100% | 8,00E-87 | 59.3% | 4XCS_A |
| Peroxiredoxin-1 *(H. sapiens*) | 256 | 256 | 100% | 2,00E-86 | 59.3% | NP_001189360.1 |
| Peroxiredoxin-1 isoform X1 (*C. lupus familiaris*) | 255 | 255 | 100% | 5,00E-86 | 58.79% | XP_038543111.1 |
| Peroxiredoxin-1 (*C. lupus familiaris*) | 254 | 254 | 100% | 6,00E-86 | 58.79% | NP_001239094.1 |
| Unnamed protein product (*H. sapiens*) | 252 | 252 | 100% | 6,00E-85 | 58.79% | BAG34839.1 |
|  |  |  |  |  |  |  |
| **Uncharacterized protein** | | | | | | |
| **Name** | **Max score** | **Total score** | **Query cover** | **E value** | **Per. Ident.** | **Accession** |
| Conserved hypothetical protein (*L. mexicana* MHOM/GT/2001/U1103) | 693 | 693 | 100% | 0.0 | 100% | XP_003872211.1 |
| Conserved hypothetical protein (*L. infantum* JPCM5) | 684 | 684 | 100% | 0.0 | 98.81% | XP_001463340.1 |
| Conserved hypothetical protein (*L. major* strain Friedlin) | 681 | 681 | 100% | 0.0 | 98.21% | XP_001680971.1 |
| Hypothetical protein LPMP 070690 (*L. panamensis*) | 660 | 660 | 100% | 0.0 | 93.73% | XP_010704077.1 |
| Conserved hypothetical protein (*L. braziliensis* MHOM/BR/75/M2904) | 659 | 659 | 100% | 0.0 | 93.43% | XP_001562567.1 |
|  |  |  |  |  |  |  |
| **Name** | **Max score** | **Total score** | **Query cover** | **E value** | **Per. Ident.** | **Accession** |
| Hypothetical protein MOQ TM35 008275 (*T. cruzi marinkellei*) | 196 | 196 | 100% | 4,00E-59 | 33.43% | EKF27989.1 |
| Hypothetical protein, conserved (*T. brucei brucei* TREU927) | 163 | 163 | 100% | 2,00E-46 | 34.1% | XP_846954.1 |
|  |  |  |  |  |  |  |
| Non significant similarity found when Hypothetical protein was compared with *H. sapiens* and *C. lupus familiaris* | | | | |  |  |
|  |  |  |  |  |  |  |
| **Kinetoplast-associated protein-like protein** | | | | | | |
| **Name** | **Max score** | **Total score** | **Query cover** | **E value** | **Per. Ident.** | **Accession** |
| Kinetoplast-associated protein-like protein (*L. infantum* JPCMS) | 878 | 878 | 100% | 0.0 | 100% | XP_001466296.1 |
| Kinetoplast-associated protein-like protein (*L. donovani*) | 853 | 58917 | 99% | 0.0 | 99.24% | AYU79903.1 |
| Kinetoplast-associated protein-like protein (*L.mexicana* MHOM/GT/2001/U1103) | 793 | 39098 | 99% | 0.0 | 96.5% | XP_003876598.1 |
| Kinetoplast-associatedprotein-like protein (*L. major* strain Friedlin) | 765 | 1.37e+05 | 99% | 0.0 | 92.24% | XP_003721830.1 |
| Kinetoplast-associated protein-like protein (*L. braziliensis* MHOM/BR/75/M2904) | 660 | 11019 | 95% | 0.0 | 88.04% | XP_001565786.1 |
| Kinetoplast-associated protein-like protein (*L. panamensis*) | 123 | 5612 | 95% | 1,00E-32 | 79.82% | XP_010700112.1 |
|  |  |  |  |  |  |  |
| **Name** | **Max score** | **Total score** | **Query cover** | **E value** | **Per. Ident.** | **Accession** |
| Kinetoplast DNA-associated protein (*T. cruzi* strain CL Brener) | 321 | 5649 | 98% | 9,00E-94 | 61.04% | XP_806768.1 |
| Hypothetical protein conserved (*T. brucei brucei* TREU927) | 68.9 | 396 | 93% | 7,00E-11 | 29.67% | XP_844266.1 |
| Putative kinetoplast DNA-associated protein (*T. cruzi*) | 94.7 | 881 | 80% | 7,00E-19 | 79.26% | PWV12676.1 |
|  |  |  |  |  |  |  |
| **Name** | **Max score** | **Total score** | **Query cover** | **E value** | **Per. Ident.** | **Accession** |
| Plectin 1, intermediate filament binding protein 500kDą, isoform CRA c (*H. sapiens*) | 55.1 | 102 | 57% | 2,00E-06 | 40.23% | EAW82172.1 |
| Plectin isoform X5 (*C. lupus familiaris*) | 52.8 | 100 | 57% | 1,00E-05 | 42.19% | XP_038542226.1 |
|  |  |  |  |  |  |  |
| **Putative heat shock protein DNAJ** | | | | | | |
| **Name** | **Max score** | **Total score** | **Query cover** | **E value** | **Per. Ident.** | **Accession** |
| Putative heat shock protein DNAJ (*L. major* strain Friedlin) | 814 | 814 | 100% | 0.0 | 100.0% | XP_003722052 1 |
| Putative heat shock protein DNAJ (*L. infantum* JPCM5) | 805 | 805 | 100% | 0.0 | 98.74% | XP_001466474.1 |
| Putative heat shock protein DNAJ (*L. mexicana* MHOM/GT/2001/U1103) | 801 | 801 | 100% | 0.0 | 97.73% | XP_003876821.1 |
| Heat shock protein DNAJ, putative (*L. panamensis*) | 728 | 728 | 100% | 0.0 | 91.41% | XP_010700329.1 |
| Putative heat shock protein DNAJ (*L. braziliensis* MHOM/BR/75/M2904) | 728 | 728 | 100% | 0.0 | 91.41% | XP_001566014.1 |
| Heat shock protein DNAJ putative GeneDB:LmjF.27.2400 (*L. donovani*) | 514 | 514 | 63% | 0.0 | 98.41% | CAC5431366.1 |
| Putative DNAJ protein (*L. major* strain Friedlin) | 351 | 351 | 92% | 1,00E-117 | 47.57% | XP_001682932 1 |
| DNAJ protein putative (*L. guyanensis*) | 342 | 342 | 88% | 4,00E-114 | 47.9% | CCM15394 1 |
|  |  |  |  |  |  |  |
| **Name** | **Max score** | **Total score** | **Query cover** | **E value** | **Per. Ident.** | **Accession** |
| Chaperone DNAJ protein putative (*T. cruzi marinkelleil*) | 555 | 555 | 100% | 0.0 | 67.66% | EKF26575.1 |
| Chaperone protein DNAJ putative (*T. brucei brucei* TREU927) | 509 | 509 | 93% | 2,00E-180 | 66.22% | XP_951689.1 |
|  |  |  |  |  |  |  |
| **Name** | **Max score** | **Total score** | **Query cover** | **E value** | **Per. Ident.** | **Accession** |
| DnaJ homolog subfamily A member 4 (*C. lupus familiaris*) | 319 | 319 | 97% | 7,00E-105 | 45.69% | XP_038333248.1 |
| Dnaj homolog subfamily A member 4 isoform 2 (*H. sapiens*) | 314 | 314 | 97% | 2,00E-103 | 45.82% | NP_001123654.1 |
|  |  |  |  |  |  |  |
